# Histone N-tails modulate sequence-specific positioning of nucleosomes

**DOI:** 10.1016/j.jbc.2024.108138

**Published:** 2024-12-26

**Authors:** Tatiana Nikitina, Wilfried M. Guiblet, Feng Cui, Victor B. Zhurkin

**Affiliations:** 1 National Cancer Institute, National Institutes of Health, Bethesda, Maryland, USA; 2 Thomas H. Gosnell School of Life Sciences, Rochester Institute of Technology, Rochester, New York, USA

**Keywords:** nucleosome, epigenetics, histone modification, DNA sequencing, DNA structure, DNA endonuclease, electrostatics, structure-function

## Abstract

Spatial organization of chromatin is essential for cellular functioning. However, the precise mechanisms governing sequence-dependent positioning of nucleosomes on DNA remain unknown in detail. Existing algorithms, considering the sequence-dependent deformability of DNA and its interactions with the histone globular domains, predict rotational setting of only 65% of human nucleosomes mapped *in vivo*. To uncover additional factors responsible for the nucleosome positioning, we analyzed potential involvement of the histone N-tails in this process. To this aim, we reconstituted the H2A/H4 N-tailless nucleosomes on human BRCA1 DNA (∼100 kb) and compared their positions and sequences with those of the wild-type nucleosomes. We found that removal of the histone N-tails promoted displacement of the predominant positions of nucleosomes, accompanied by redistribution of the AT-rich and GC-rich motifs in nucleosome sequences. Importantly, most of these sequence changes occurred at superhelical locations (SHLs) ±4, ±1, and ± 2, where the H2A and H4 N-tails interact with the DNA minor grooves. Furthermore, a substantial number of H4-tailless nucleosomes exhibit rotational settings opposite to that of the wild-type nucleosomes, the effect known to change the topological properties of chromatin fiber. Thus, the histone N-tails are operative in the selection of nucleosome positions, which may have wide-ranging implications for epigenetic modulation of chromatin states.

Organization of eukaryotic DNA in chromatin is essential for many cellular processes such as gene expression, DNA replication and repair (1). The basic repeating unit of chromatin is the nucleosome core particle (NCP) containing ∼145 bp fragment of DNA tightly wrapped around histone octamer (2). Packaging of DNA into nucleosomes is defined by numerous factors including the DNA bending anisotropy (3, 4, 5) and interactions with histones (5, 6, 7), both of which are sequence dependent. The ATP-dependent chromatin remodeling enzymes and transcription factors can also determine nucleosome positioning patterns (8).

Histone interactions with DNA involve both the histone globular domains and the tails. For the histone domain-DNA interactions, previous studies have identified 14 highly conserved “sprocket” arginine residues deeply buried in the DNA minor groove (5, 9, 10). We use this notation (5) because the spatial arrangement of these arginines in the minor groove of nucleosomal DNA resembles the teeth of a sprocket meshing with the holes in the links of a bicycle chain. As to the histone tails, they engage in fuzzy interactions with nucleosomal DNA, which are largely electrostatic in nature (11, 12). By contrast with the “sprocket” arginines, the histone tails are highly dynamic and undergo transitions between various conformations. These transitions may be influenced by several factors such as nucleosome spacing, histone post-translational modifications (PTMs), and interactions with chromatin-associated proteins.

The histone tails (and especially the N-tails) contain numerous lysine and arginine residues that serve as a platform for PTMs (13). Epigenetic alterations of these residues can lead to changes in chromatin states (14). For example, the acetylation of lysine residues neutralizes positive charges on histones, which weakens the interaction between histone N-tails with negatively charged DNA phosphate groups. As a result, a closed chromatin state can be transformed into an open state with increased DNA accessibility. Recent studies have further elucidated this mechanism, revealing that chromatin regulation might occur through co-recognition of nucleosomal H3K4me3 and H3K14ac by the tandem reader domains. This represents a refined “histone code” concept, in which a combinatorial readout of two distinct PTMs occur within a single nucleosome (15). Note, however, that there are no published data on the local repositioning of nucleosomes caused by PTMs.

Sequence-dependent bending anisotropy dictates how DNA is wrapped around a histone octamer (3, 4, 5). Previous studies have established various DNA sequence patterns favorable for nucleosome formation (16, 17, 18, 19, 20, 21, 22, 23, 24, 25, 26, 27), including the well-known WW/SS pattern, see Figure 1. (Here and below, W stands for A or T, and S is for G or C.) Specifically, the SS dinucleotides are enriched in the positions where DNA bends into the major groove, while the WW dinucleotides are predominantly located in the sites where nucleosomal DNA bends into the minor groove. The “sprocket” arginines penetrating minor groove at these sites, interact favorably with AT-rich DNA having electro-negative minor groove (5). These energetically favorable arginine-DNA contacts that occur every 10 to 11 bp in NCPs, provide a structural basis for the rotational positioning of the WW/SS nucleosomes.

**Figure 1:**
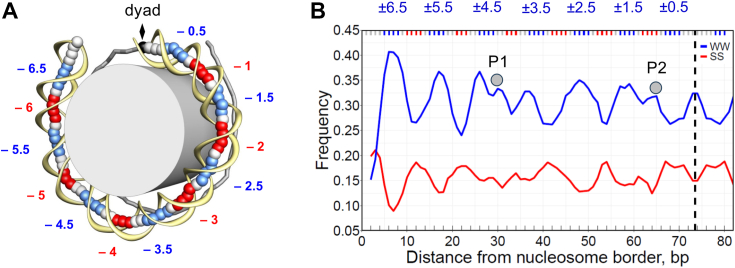
**Locations of the minor- and major-groove bending sites and distributions of WW and SS dinucleotides along nucleosomal DNA.***A*, the crystal structure of the 1k × 5 nucleosome core particle with 147-bp long DNA (32) shown schematically: the DNA fragment is divided into two halves, separated by the dyad (diamond). The base-pair centers in the ‘ventral’ half are represented by large balls, and the sugar-phosphate backbone is shown by *yellow* ribbon. In the ‘dorsal’ half of nucleosome, sticks connect the base-pair centers. (Only small fragments of the stick representations are visible to the reader.) The minor- and major-groove bending sites are shown in *blue* and *red*, respectively. These sites are denoted according to their superhelical locations (SHL). *B*, the frequencies of occurrences of combined AA, TT, AT, and TA dinucleotides (WW, shown in *blue*) and GG, CC, GC and CG dinucleotides (SS, shown in red) in each nucleosomal position, which were ‘symmetrized’ with respect to the dyad (*dashed line*). The three base-pair running averages of the WW and SS frequencies are presented for chicken nucleosomal DNA (27, 33). *Gray circles* indicate positions of the off-peaks, P1 and P2. The superhelical locations are shown above the plot (see also Figure 2, Figure 3, Figure 4).

In addition, the alternative DNA sequence pattern known as anti-WW/SS was described, in which the WW and SS profiles are in counterphase with the corresponding profiles observed in canonical WW/SS nucleosomes (28, 29). In this case, the “sprocket” arginines seem to be in unfavorable contacts with GC-rich fragments in minor groove. Nevertheless, a recent study showed that the anti-WW/SS nucleosomes are widespread across different eukaryotes accounting for ∼25% in mammalian genomes (29). It remains unclear how these nucleosomes are stabilized. Note that other sequence patterns were also described (28), including RR/YY and anti-RR/YY pattern (where R is A or G and Y is C or T). Nucleosomes with the RR/YY pattern are frequently found in promoter regions, but not in other genomic regions (28). While nucleosomes with the RR/YY and related patterns may have implications in gene regulation, their analysis is beyond the scope of the present study.

Comparing several algorithms predicting positioning of human nucleosomes *in vivo* (30), we showed that our algorithm (analyzing distribution of flexible pyrimidine-purine YR dimers as well as the WW/SS motifs) predicted 65% of the positions with 2-bp precision, whereas the widely used Kaplan-Segal model (31) predicted 55% of the nucleosomal positions. In other words, 35 to 45% of nucleosome positions observed *in vivo* remain unaccounted for.

To tackle this problem, we analyzed the distributions of mono- and dinucleotides (A, T, W, WW, and SS) along nucleosomal DNA across different eukaryotic genomes (Figs. 1*B* and 2). In addition to the periodically oscillating sequence patterns observed earlier (27), we found two sets of ‘rogue’ signals, one near superhelical locations (SHL) ±4 and the other near SHL ±1. Note that in published nucleosome structures (2, 32) the lysine-rich N-tails of the H2A and H4 histones are in favorable contact with the AT-rich DNA minor groove at SHL ±4 and SHL ±1/±2, respectively (Fig. S1). Therefore, we hypothesized that the histone tails may play a role in the process of sequence-driven selection of the optimal nucleosome positions on DNA.

**Figure 2:**
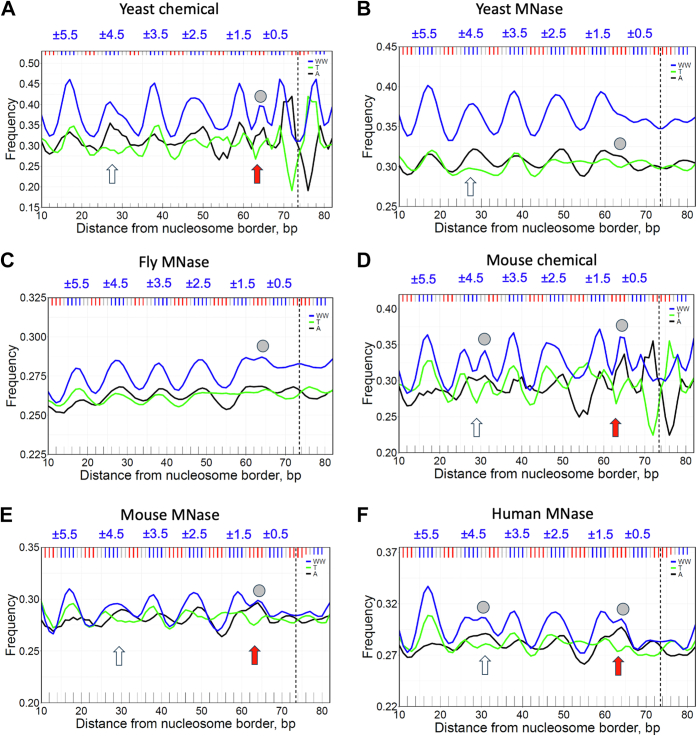
**The frequencies of occurrences of nucleotides A and T as well as dinucleotide WW in yeast (A, B), fly (C), mouse (D, E) and human (F) nucleosomal DNA.** The yeast and mouse nucleosomes are represented by two datasets, obtained with chemical mapping (*A*, *D*) and with MNase-seq mapping (*B*, *E*). The off-peaks, P1 and P2, are marked by *gray circles* (see Fig. 1*B*). *Arrows* indicate the DNA regions with strand-specific preference A > T (SHL ±4.5, ±1). Note that at SHL ±1 the A > T effect is the strongest for mouse and human.

To elucidate the possible effect of the H2A and H4 N-tails on the sequence-specific formation of nucleosomes, we reconstituted the N-tailless NCPs and compared their positioning *in vitro* with that of the wild type (wt) NCPs. We found that overall, the H2A N-tailless NCPs are positioned in phase with the wt-NCPs (*i.e*., they are shifted by 10*n* bp, where *n* = 0, 1, 2, …). By contrast, a substantial number of the H4 N-tailless NCPs are in counterphase with the wt-NCPs (*i.e*., they are shifted by 10*n* + 5 bp, like the anti-WW/SS nucleosomes are shifted *versus* canonical nucleosomes). Thus, our results suggest that preferential interactions of the histone N-tails with AT-rich minor groove may provide a novel mechanism for stabilization of anti-WW/SS nucleosomes observed *in vivo*. Implications for epigenetic control of nucleosome positioning are also discussed.

## Results

### Periodically oscillating sequence motifs in nucleosomal DNA

The periodically oscillating nucleosomal DNA sequence motifs have been observed in the pioneer study by Satchwell, Drew and Travers (27) who analyzed distributions of all dimeric and trimeric steps in chicken nucleosomes *in vivo*. Their observation that the AT-rich and GC-rich motifs preferentially bend into the minor and major groove, respectively, was confirmed by numerous studies (33, 34, 35). These collective efforts have led to the widely accepted WW/SS scheme which we are using to represent the sequence-dependent deformability of nucleosomal DNA (Fig. 1*B*). Note that the maxima of the WW profile occur in the minor-groove bending sites (SHL ±6.5, ±5.5, ±4.5, ±3.5, ±2.5, ±1.5), whereas the SS maxima occur in the major-groove bending sites (SHL ±6, ±5, ±4, ±3, ±2) (Fig. 1*B*). Besides the periodically oscillating WW peaks, two pairs of distinctive ‘off-peaks’ P1 and P2 are clearly visible (Fig. 1*B*), one near SHL ±4 (P1) and the other at SHL ±1 (P2). Since no distortion in DNA trajectory is observed at these sites in nucleosome structures (2, 32), potentially, the irregularities in the WW profile may indicate the existence of certain histone-DNA interactions, in addition to those involving the ‘sprocket’ arginine residues (5, 6, 7, 9, 10).

To elucidate the sequence specificity of these off-peaks, we calculated the high-resolution profiles of mononucleotides A and T as well as the dinucleotide WW in nucleosomal DNA obtained from various species (see Fig. 2, where the off-peaks P1 and P2 are marked by grey circles). Here we consider only 147-bp nucleosomal DNA fragments selected from millions of mapped NCPs, thereby increasing resolution of the sequence profiles, compared with the early studies (27). Note that in addition to the chicken MNase dataset (Fig. 1*B*), the off-peak P1 is observed in the mouse chemical and the human MNase datasets (Fig. 2, *D* and *F*), while the off-peak P2 is present in all datasets. In most cases, it is detectable in the WW and A profiles, except the yeast MNase where it is clearly visible only in the A profile. Thus, we see that the off-peaks P1 and P2 are not an artefact observed only in a single species or after specific treatment (*e.g*., as a result of MNase or chemical cleavage); rather, they are inherent in a wide spectrum of eukaryotes.

Both off-peaks P1 and P2 have a clear strand-specific preference for A *versus* T (see arrows in Fig. 2). This effect was described by us earlier for yeast (35). The A > T preference at SHL ±4 (P1) was attributed to wedge formation by AA:TT dimeric steps observed in this region of alpha-satellite nucleosomal DNA crystal structure (32). According to this interpretation, the AA:TT wedges (18, 23) facilitate anisotropic DNA bending in nucleosome (4, 19), therefore, the AA dimers are more abundant in the leading strand at this location. This explanation is not universal because it does not account for the off-peak P2 at SHL ±1.

Note, however, that the locations SHL ±4 and SHL ±1 (where the off-peaks P1 and P2 are positioned) coincide with locations where the N-tails of H2A and H4 histones are in close contact with the DNA minor groove (Fig. S1). Since the nucleosomal DNA is relatively AT-rich (and depleted of the guanine amino groups) at these locations, its minor groove is electro-negative and attractive for the positively charged histone N-tails. Hence, we posit that the histone N-tails may be responsible, at least partially, for formation of the off-peaks P1 and P2 (characterized by an increased occurrence of WW dimers).

### Histone H2A N-tailless constructs *in vitro*

**Figure 3:**
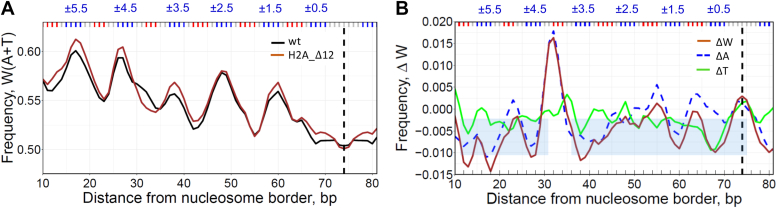
**DNA sequence analysis of nucleosomes reconstituted with H2A N-tailless histones.***A*, the frequencies of occurrence of W(A + T) nucleotides in the wt (*black*) and H2A_Δ12 nucleosomes (*brown*). Three base-pair running averages of the frequencies were plotted over each nucleosomal position and symmetrized with respect to the nucleosome dyads (dashed lines). *B*, differential ΔW, ΔA, and ΔT profiles (*brown*, broken *blue*, and *green lines*, respectively). The ΔW values were calculated as the W(A + T) frequency in wt nucleosomes minus frequency in H2A_Δ12 nucleosomes for each nucleosomal position. The shaded areas represent the average ΔW = −0.006 and standard deviation of ΔW (0.004) in the regions 10 to 29 and 35 to 74.

To examine the role of histone N-tails in the selection of optimal nucleosomal DNA sequences *in vitro*, we compared the local AT content in nucleosomes reconstituted with the wild type and the H2A N-tailless histones (H2A_Δ12 with 12 amino acids truncated at the N-end, see Methods). First, we analyzed the combined frequencies of occurrences of adenines and thymines in the wt-nucleosomes and H2A_Δ12 constructs (Fig. 3*A*) and found that both W(A + T) profiles exhibit periodic patterns with the peaks around the minor-groove bending sites, similar to the WW pattern of nucleosomes *in vivo* (Figs. 1 and 2). Overall, the AT content in the wt-nucleosomes is lower by 0.4%, except for the narrow interval near position #30 where the AT content is higher by ∼2%.

To better visualize the changes in sequence composition between the two populations of NCPs, we calculated ΔW value representing the change in W(A + T) frequency upon transition from the N-tailless to the wt-nucleosomes (Fig. 3*B*). The differential ΔW profile further demonstrates that the changes in W(A + T) are local, that is, the AT content has increased noticeably in the region #30 to 34 (where H2A tail interacts with the DNA minor groove in X-ray structure (32), while in the other NCP regions the 'compensatory' changes in ΔW profile are relatively minor and exhibit elements of randomness (Fig. 3*B*).

Importantly, the local increase in the AT-content occurs exclusively due to adenines (Fig. 3*B*), which is entirely consistent with the strand-specific preference for A *versus* T described above *in vivo* (see Fig. 2*F*, position #30). Note that the increase in the fraction of adenines at positions #31 to 33 is statistically significant (Fig. S2). This result proves that indeed, modification of the histone H2A N-tails (in particular, their truncation) produces detectable changes in the W(A + T) frequency profile of the NCPs reconstituted *in vitro.* As shown below, these changes are accompanied by nucleosome repositioning (Fig. S3).

Above, we analyzed the DNA fragments obtained as a result of MNase cleavage of the reconstituted nucleosomes. To exclude the sequence bias due to the known AT-specificity of micrococcal nuclease (36) we repeated the same procedure using combined MNase and ExoIII digestion of nucleosomes (35, 37). Remarkably, both approaches gave practically the same result (compare Figs. S4 and 3*B*). That is, the ΔW profile of the MNase-Exo data displayed a single strong peak at positions #31 to 32, predominantly made up of adenines, which was significantly higher than ΔW values at other positions. This consistency between the MNase and MNase-Exo data further supports our conclusion that the H2A N-tail interacts with nucleosomal DNA in a sequence-specific manner.

### Histone H4 N-tailless constructs *in vitro*

**Figure 4:**
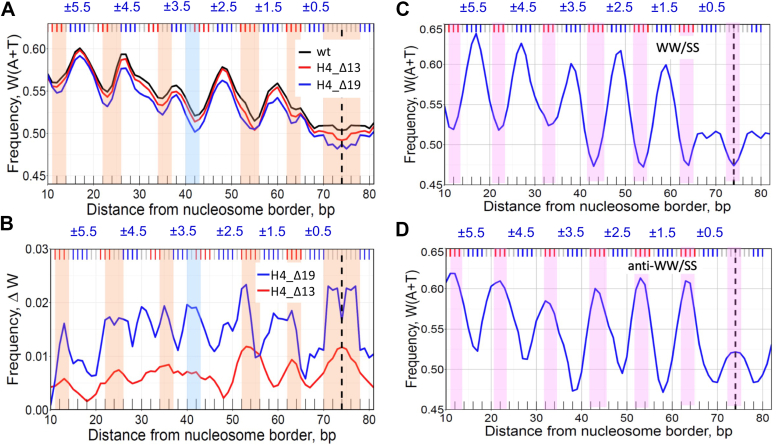
**DNA sequence analysis of nucleosomes reconstituted with H4 N-tailless histones.***A*, the frequencies of occurrence of W(A + T) nucleotides in the wt-nucleosomes (*black*), H4_Δ13 (*red*) and H4_Δ19 (*blue*). *B*, differential ΔW profiles for H4_Δ13 (*red*) and H4_Δ19 (*blue*) nucleosomes. The ΔW values were calculated as in Figure 3. The *orange* vertical stripes emphasize close correspondence between the local minima in W(A + T) profile (*A*) and the local maxima in ΔW (*B*). The *light blue* vertical stripe indicates the local minima in W(A + T) at position #42 that have no corresponding local maximum in ΔW (H4_Δ13). *C* and *D*, W(A + T) profiles for the wt-nucleosomes having the canonical WW/SS pattern (*C*) and the anti-WW/SS pattern (*D*). See ref. (29) for the description of the detailed procedure. The two populations of nucleosomes presented in (*C*, *D*) were denoted as Type 1 and Type 4, respectively (29). The pink vertical stripes indicate local minima in the W(A + T) profile for nucleosomes having the WW/SS pattern (*C*), and local maxima for nucleosomes having the anti-WW/SS pattern (*D*). Note that anti-WW/SS nucleosomes comprise 23% of all nucleosomes *in vitro*, in close agreement with the ∼25% fraction of human nucleosomes *in vivo* (29).

We studied two H4 mutants, H4_Δ13 and H4_Δ19, with 13 and 19 amino acids truncated at the N-end (see Methods). Removal of the H4 N-tail produced quite a different effect compared to H2A (Fig. 4*A*). Instead of the local changes in W(A + T) frequency observed for the H2A_Δ12 construct, in the case of H4_Δ13 and H4_Δ19, the AT-content has decreased by 1 to 2% (relative to the wild-type level) all over the length of a nucleosome. This is clearly visible in the differential ΔW profiles (Fig. 4*B*). For the H4_Δ13 mutant, ΔW exhibits strong peaks in the central part of nucleosome, at SHL 0 (dyad), SHL ±1, and ± 2. For the H4_Δ19 construct, the ΔW peaks are approximately two times higher and more widespread (Fig. 4*B*). In other words, a complete truncation of N-tail (in H4_Δ19) leads to the more pronounced changes in the W(A + T) profile of nucleosome DNA sequences, compared to the partial removal of this tail (in H4_Δ13).

Direct comparison of the ΔW and W(A + T) profiles shows that they are negatively correlated (Fig. 4, *A* and *B*). Indeed, each local minimum in W(A + T) corresponds to one of the local maxima in ΔW (H4_Δ19), as shown by vertical stripes in Figure 4. In the case of H4_Δ13, six out of seven local minima in W(A + T) correspond to one of the local maxima in ΔW (H4_Δ13). (The only exception is position #42; see the light blue vertical stripe.) In other words, the ΔW (H4) values are the highest where W(A + T) are the lowest. Thus, we observe two periodically oscillating signals in the nucleosomal DNA sequences – in addition to the canonical W(A + T) pattern, there is the ΔW pattern, associated with the H4 N-tail and positioned in counterphase with W(A + T).

In this regard, note that the ΔW (H4_Δ19) profile resembles the anti-WW/SS profile studied earlier (29) − both have peaks at the major-groove bending sites (Fig. 4, *B* and *D*), which is the opposite of the canonical W(A + T) profiles (Fig. 4, *A* and *C*). The rotational settings of the WW/SS and anti-WW/SS nucleosomes are counter-phased -- that is, the relative shift between these nucleosomes is ∼10*n* + 5 bp (5, 15, 25, *etc.*). Therefore, one can expect that a similar shift of ∼10*n* + 5 bp would be observed between the wild type and H4-tailless nucleosomes (Fig. 4, *A* and *B*).

### Inter-nucleosome distance cross-correlation

**Figure 5:**
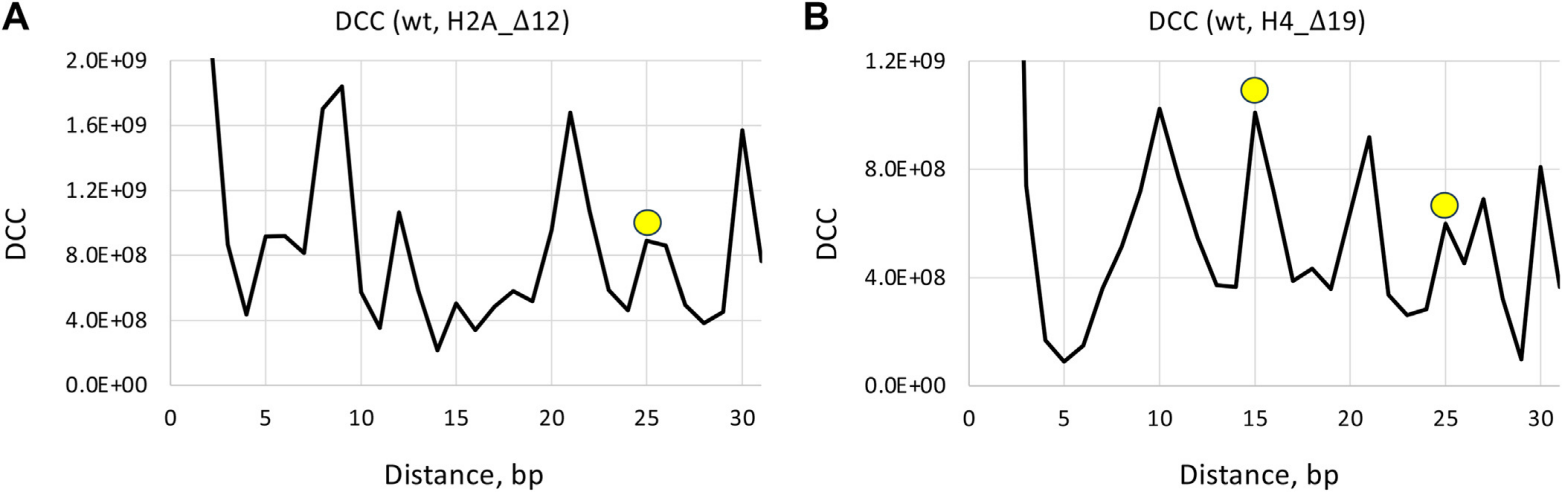
**Inter-nucleosome distance cross-correlation functions (DCC).***A*, cross-correlation between NCP positions in the wild type and H2A_Δ12 datasets. *B*, cross-correlation between NCP positions in the wild type and H4_Δ19 datasets. The peaks at 15 and 25 bp are emphasized by *yellow circles*. The NCP positions with occurrences > 5000 were included in the calculation of DCC (see Figs. S3, S5 for typical occurrence values).

To describe the relative positions of nucleosomes in two populations we used the distance cross-correlation (DCC) function introduced earlier (34). For each pair of NCP positions (one from the first set, and the other from the second set), the distance ‘*dist*’ between the NCP dyads is calculated, and the occurrences of all distances are summed up (Fig. 5). Note that multiple occurrences of nucleosomes in the same position are counted multiple times, that is, if the two NCP dyad positions are separated by 20 bp and occur 5 and 10 times, respectively, the corresponding DCC (*dist* = 20) is calculated as 5 × 10 = 50.

A comparison of the wt and H2A-tailless nucleosome subsets revealed strong DCC peaks at ∼10, ∼20 and 30 bp (Fig. 5*A*), indicating that the NCPs from wt- and H2A_Δ12-subsets are mostly separated by an integral number of DNA helical turns and represent the rotationally related translational positions (34, 38); see Fig. S3 where the wt and H2A_Δ12 nucleosomes are shifted by 20 bp.

In the case of wt and H4_Δ19 nucleosomes, the DCC profile has an additional strong peak at 15 bp (Fig. 5*B*), suggesting that there are quite a few occurrences when nucleosomes from the two subsets have opposite rotational settings. The representative example is given in Fig. S5, where the NCP occurrence profiles are shown for the 1 kb region of the BRCA1 gene. Note that both profiles have twin peaks separated by 15 bp. In the wt-set, the dominant NCP position corresponds to the 'left' peak, whereas in the H4_Δ19 set, the dominant NCP position coincides with the “right” peak (Fig. S5, *A* and *B*). Furthermore, the H4_Δ13 nucleosomes follow the same trend as H4_Δ19 (Fig. S5*C*), and H2A nucleosomes are positioned similarly to wt-nucleosomes (Fig. S5*D*). We consider this result as proof of principle demonstrating that predominant NCP positions in the wt and H4-tailless populations can be separated by 10*n* + 5 bp (that is, can have opposite rotational settings).

### A novel mechanism for stabilization of the anti-WW/SS nucleosomes

**Figure 6:**
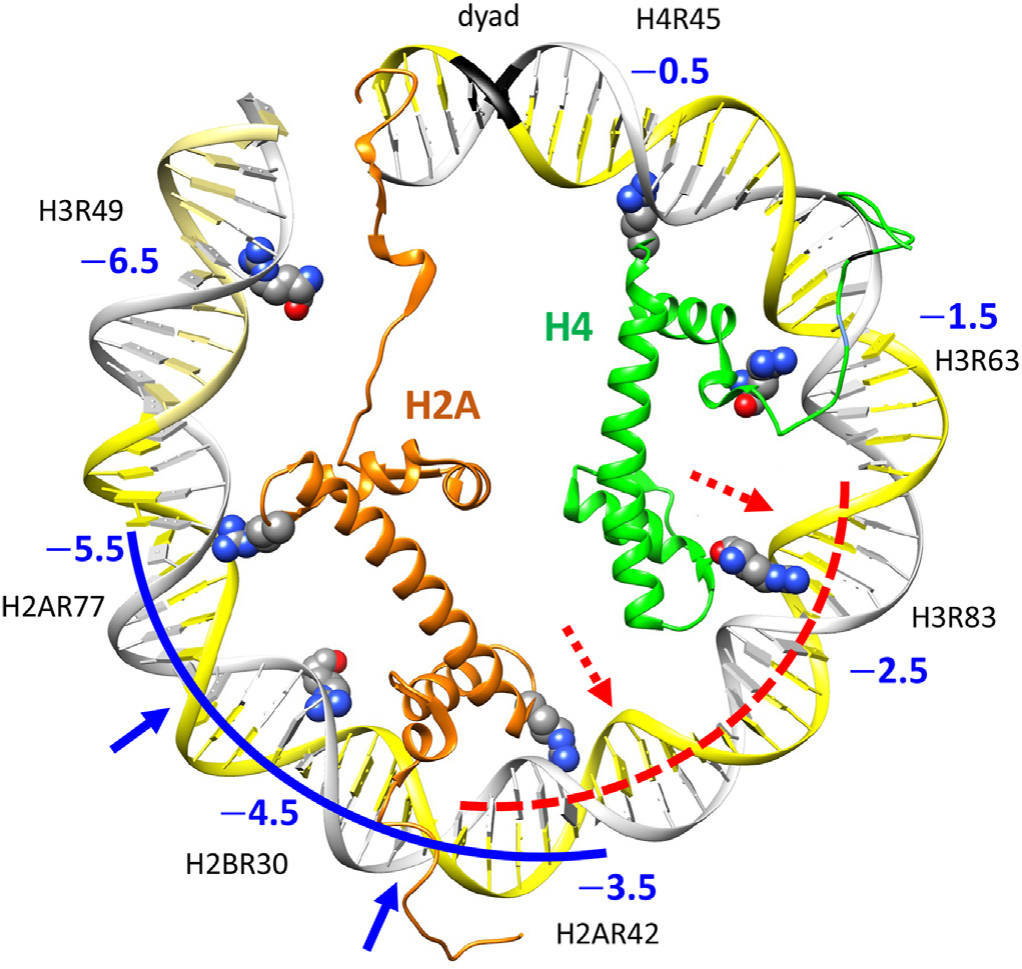
**Two types of sequence-specific histone-DNA interactions.** Interactions of the first type (with the “sprocket” arginines) stabilize 'canonical' nucleosomes with the WW/SS sequence pattern (Fig. 1). Interactions of the second type (with the H2A and H4 N-tails) are favorable for nucleosomes with the anti-WW/SS sequence pattern (28, 29). Both types of histone-DNA interactions can co-exist in the same nucleosome. The ventral half of the nucleosome 1k × 5 (32) is shown (the DNA sites from SHL -6.5 to SHL -0.5). The seven “sprocket” arginines interact with DNA at locations where the minor groove is facing in (3, 4, 5). Only histones H2A (*orange*) and H4 (*green*) are shown, with their N-tails penetrating the DNA minor grooves close to SHL -4.5 and SHL -1.5, respectively. The dorsal half of nucleosome 1k × 5 (with the positive SHL values) is presented in Fig. S1*B*. The nucleosome positions optimal for interactions with the “sprocket” arginines and the histone N-tails are not necessarily the same. In the case of H4 N-tail, these positions are often separated by 10n + 5 bp, which means that they have opposite rotational settings (see Figs. 5*B* and S5 where the two NCP positions are separated by 15 bp). This effect has important implications for the transcription factors binding nucleosomes (*e.g*., pioneer factors). For illustration, consider the tumor suppressor protein p53, whose consensus binding site comprises the 20-bp DNA fragment (67). In one of the two NCP positions, the p53 20-mer spans the interval from SHL -5.5 to SHL -3.5, being in an exposed configuration suitable for the p53 binding (see the *blue* curve and arrows). Whereas in the alternative NCP position, the p53 20-mer is shifted by 15 bp, so that its rotational setting is changed by ∼180° (compare the two 20-mers outlined by the *red* and *blue* curves). Consequently, the '*red*' p53 20-mer is in the hidden configuration and the p53 protein cannot bind to this site due to steric repulsion with the histone octamer (broken *red* arrows). This is just one example suggesting that the NCP shift by 10*n* + 5 bp (caused by abrogation of the histone N-tail binding to DNA) can play the role of a conformational switch between the hidden and exposed configurations of the protein-binding sites integrated in nucleosomes.

According to our model, a nucleosome is stabilized by two types of sequence-specific histone-DNA interactions. The first type comprises the ‘sprocket’ arginines in histone globular domains penetrating the DNA minor groove at locations SHL ±0.5, ±1.5, ... ±6.5 (3, 4, 5), see Figure 6. These locations are characterized by a narrowed minor groove and are usually enriched with the WW dinucleotides (Fig. 1). The positively charged arginines thus favorably interact with the electro-negative minor groove facing in, thereby selecting (and stabilizing) nucleosomes with the canonical WW/SS sequence pattern.

The second type of sequence-specific interactions involves the H2A and H4 histone N-tails, which are in close contact with the minor groove facing out at SHL ±4 and SHL ±2/±1, respectively (Figs. 6, S1). Our data indicate that the wt-nucleosomes containing N-tails are enriched with the AT-containing fragments at these sites. This trend is opposite to the WW/SS sequence pattern but is consistent with the anti-WW/SS pattern (Fig. 4*D*). Therefore, we suggest that the anti-WW/SS nucleosomes are stabilized (at least partially) by the H2A and H4 histone N-tails favorably interacting with the AT-rich minor groove because they are enriched by lysines and arginines. This interpretation is consistent with the NMR data and MD simulations showing that the minor groove conformer of the H2A N-tail is likely to interact with DNA stronger than its major groove conformer (39). Schematically, the H2A and H4 N-tails act like the arms embracing nucleosomal DNA from outside, whereas the ‘sprocket’ arginines act like the hooks holding nucleosomal DNA from inside (5).

The two types of histone-DNA interactions may be involved in a tug-of-war, as shown in Fig. S5. Depending on the presence or absence of the histone N-tails, the optimal positioning of a nucleosome corresponds to one of the two opposite rotational settings (that is, the two NCP positions are separated by 10*n* + 5 bp). This effect has important implications, both locally and globally. Locally, the NCP shift by 10*n* + 5 bp implies a conformational switch between the hidden and exposed configurations of the protein-binding sites (embedded into the nucleosomal sequence). This is illustrated in Figure 6 in the case of the tumor suppressor protein p53. Globally, the nucleosome sliding by 10*n* + 5 bp brings about significant changes in the topological state of the chromatin fiber (40, 41), mostly due to the changes in DNA writhing (42).

Note that in our *in vitro* experiments, the histone N-tails are truncated, while *in vivo* a similar effect is produced by PTMs of lysines and arginines, resulting in a (partial) abrogation of the electrostatically favorable interactions between the histone tails and the DNA minor grooves. Hence, we anticipate that the simple scheme presented above applies to the mechanisms of epigenetic regulation of nucleosome positioning *in vivo.*

## Discussion

Our study was initiated, in part, by the earlier observations of the ‘rogue’ signal near SHL ±1 by Davey (43) and the A *versus* T strand asymmetry near SHL ±4 by Cole *et al.* (35). Various interpretations of these peculiar effects were considered, including interaction of arginine H3R40 with electronegative groups of A:T base pairs near position #65 (43) and wedge formation by AA:TT dimeric steps near #30 (35). However, neither of these explanations account for the position-dependent preference for A over T and T over A (*e.g*., compare positions #30 and #52, Fig. 2). It was noted (35) that "an additional factor" is required to elucidate the A *versus* T strand asymmetry in nucleosomes. Our results suggest that the interaction of the histone N-tails with the DNA minor groove is likely to be such a factor.

The dynamic nature of these interactions was clearly demonstrated by extensive molecular dynamics (MD) simulations performed by Shaytan *et al.* (44). The histone tails fluctuate in the vicinity of the DNA surface, with the DNA minor grooves serving as kinetic traps. The H2A and H4 N-tails mostly stay within the limits indicated in Fig. S1, closely resembling the x-ray structure (32). In particular, the H4 N-tails remain most of the time in the minor grooves close to SHL ±1 or SHL ±2; they do not penetrate the minor groove at SHL 0 (near the dyad), at least during the 1-microsecond simulations. The 4-microsecond MD simulations performed later with different solvent parameterization (45) gave essentially the same result – namely, the H4 N-tails don’t go beyond the limits established by the x-ray structure (32). Finally, recent NMR study (46) accompanied by 5-microsecond MD simulations has also localized the H4 tails in the DNA region from SHL ±1 to SHL ±2.5.

Note, however, that the H4 N-tail is sufficiently long to penetrate the minor groove close to SHL 0 (Fig. S1*A*). Our result on the high ΔW value in the vicinity of the dyad (Fig. 4*B*) suggests that the H4 N-tail might interact directly with DNA at SHL 0 (but this is not the only possible interpretation). It would be interesting to see if the additional increase in the time of MD simulations will allow observing the larger-scale fluctuations of the H4 N-tail.

The first observations of the nucleosome displacement related to the complete removal or modifications of the histone tails were made more than 20 years ago. Hamiche *et al.* (47) investigated nucleosome mobility using reconstituted NCPs lacking one or more histone N-tails and detected nucleosome sliding promoted by the removal of the H2B N-tail. Fan *et al.* (48) analyzed the positioning of nucleosomes containing histone H2A.Z on the sea urchin 5S RNA gene. They found that the substitution of histone H2A by H2A.Z results in a shift of the predominant nucleosome position by 20 bp, which is the same shift as observed here for the H2A N-tailless nucleosomes (Fig. S3). This similarity confirms our conclusion on the role played by the histone H2A N-tail in the selection of the translational nucleosome positioning while maintaining the same rotational setting. Furthermore, Yang *et al.* (49) analyzed positioning of tailless nucleosomes on the *Xenopus borealis* somatic-type 5S RNA gene and also observed the 20-bp shift. Formally speaking, it would be incorrect to compare directly this 20-bp shift with our result, because Yang *et al.* digested by trypsin all histone tails (not only the H2A N-tail). Nevertheless, we can safely summarize that the removal of histone tails can lead to a noticeable alteration of the sequence-dependent nucleosome positioning *in vitro.*

On the other hand, *in vivo*, the clipping of histone tails by proteases plays a role in the regulation of stem cell differentiation (50) and other cellular processes (51, 52). Therefore, it is reasonable to assume that the repositioning of the N-tailless nucleosomes observed here may (at least, partially) account for induction of transcription produced by enzymatic cleavage of the histone N-tails (53).

Furthermore, histone tails have been shown to form reversible DNA-binding crosslinks with epigenetic bases such as N7-methyl-2′-deoxyguanosine, a harmful type of DNA damage resulting from DNA alkylating agents (54). Recent data indicates that histone tails can also influence the local ionic strength near nucleosomal DNA, which in turn affects the chemical reactivity of damaged DNA (55). These findings underscore the pivotal role of histone tails in this biologically important chemistry, which can directly impact nucleosome (re)positioning. Our studies extend these findings to include interactions with regular DNA nucleotides, focusing on electrostatic interactions between the histone tails and DNA.

## Conclusion

Analyzing distribution of the AT-rich fragments in nucleosomal DNA sequences *in vivo*, we observed non-canonical peaks in the vicinity of superhelical locations SHL ±4 and SHL ±1. In the nucleosome X-ray structure (32), DNA at these locations is in contact with the N-tails of histones H2A and H4, respectively. Therefore, we hypothesized that the observed irregularities in DNA sequence are associated with the favorable interactions between the AT-rich DNA and the lysine-rich histone N-tails.

To test this hypothesis, we reconstituted the H2A- and H4-tailless NCPs *in vitro* and analyzed the local changes in the AT content of these nucleosomes compared to the wt-nucleosomes. We found that the differences between sequences of H2A-tailless and wt-nucleosomes are localized around SHL ±4, while the most pronounced changes in the H4-tailless NCP sequences occur in the central part of a nucleosome (*i.e*., near dyad and at SHL ±1/±2). These results are consistent with the above assumption that the H2A and H4 N-tails are involved in selection of the optimal NCP sequences. As to the putative interactions between the H4 N-tail and DNA in the vicinity of the dyad (at SHL 0), further structural studies are needed to clarify this question.

Our main results are based on the sequence analysis of the reconstituted nucleosomes obtained after MNase cleavage, which is known to be AT-specific (36). To exclude the possible MNase-associated bias in selection of nucleosomes, we used a combined MNase and ExoIII digestion of nucleosomes (35, 37). Both approaches gave entirely consistent results (see Fig. 3*B* and S4), thereby supporting our inference on the sequence-specific interactions between the histone N-tails and nucleosomal DNA.

To summarize, our data suggest that histone tails preferentially select certain nucleosome positions over other positions, which may have important implications for epigenetic modulation of nucleosome positioning. Certain epigenetic marks, such as the acetylation of lysines on histone N-tails, decrease affinity of the tails to the DNA minor groove. This, in turn, may change nucleosome positions and even shift the equilibrium between different chromatin conformation states. Obviously, more studies are needed to elucidate the role of epigenetic modifications of histones in the positioning of nucleosomes.

## Methods and materials

### Nucleosome array reconstitution

Transformed *E. coli* with TAR/BAC containing full-length hBRCA1 gene (BRCA1-TAR/YAC/BAC) was obtained from V. Larionov (56). It was first placed on agar plates supplemented with chloramphenicol as a selective agent (0.5 ml of 25 g/ml stock solution per 1 L of LB media). Cells were transferred into filtered LB media supplemented with 10 g yeast extract, 10 g NaCl, 2 g bactotryptone, 0.5 ml of 25 mg/ml of chloramphenicol, and 0.4 ml of 5 M NaOH per 1 L of media and grown overnight at 37 °C on platform shaker. Plasmids were prepared using a Qiagen Large-construct kit (cat. # 12462) according to the manufacturer’s instruction, and then cleaned with phenol-chloroform extraction, precipitated, and resuspended in 10 mM Tris pH 8.0, 0.2 mM EDTA.

Reconstituted BRCA1-TAR/YAC/BAC arrays were assembled using *Xenopus* histone octamers with wt-histones and H2A/H4 N-tailless histones obtained from Histone Source, Protein Expression and Purification Facility at Colorado State University. Reconstitution was performed as described earlier (57). Briefly, core histone octamers and purified DNA were combined in a final mixture containing 2 M NaCl and 1 mM PMSF. Samples were kept on ice for 1 h following dialysis in 3500 MWCO membranes against buffer (2 M NaCl, 10 mM Tris, 0.2 mM EDTA, 0.1% NP-40, 5 mM β-mercaptoethanol, pH = 8.0) for 2 h. Samples were transferred to 1.5 M NaCl buffer and dialyzed for 2 h, followed by dialysis against 1 M NaCl and 0.75 M NaCl buffers, 3 h each, and 0.5 M NaCl buffer overnight (all other buffer components were kept constant). Reconstitutes were next dialyzed against a buffer containing 5 mM NaCl, 10 mM Tris, 0.2 mM EDTA, 0.1% NP-40, 5 mM β-mercaptoethanol, pH 8.0, for 3 h. Samples were dialyzed in the buffer with 5 mM NaCl, 10 mM Tris, 0.025% NP-40, and no EDTA for an additional 3 h.

**Figure 7:**
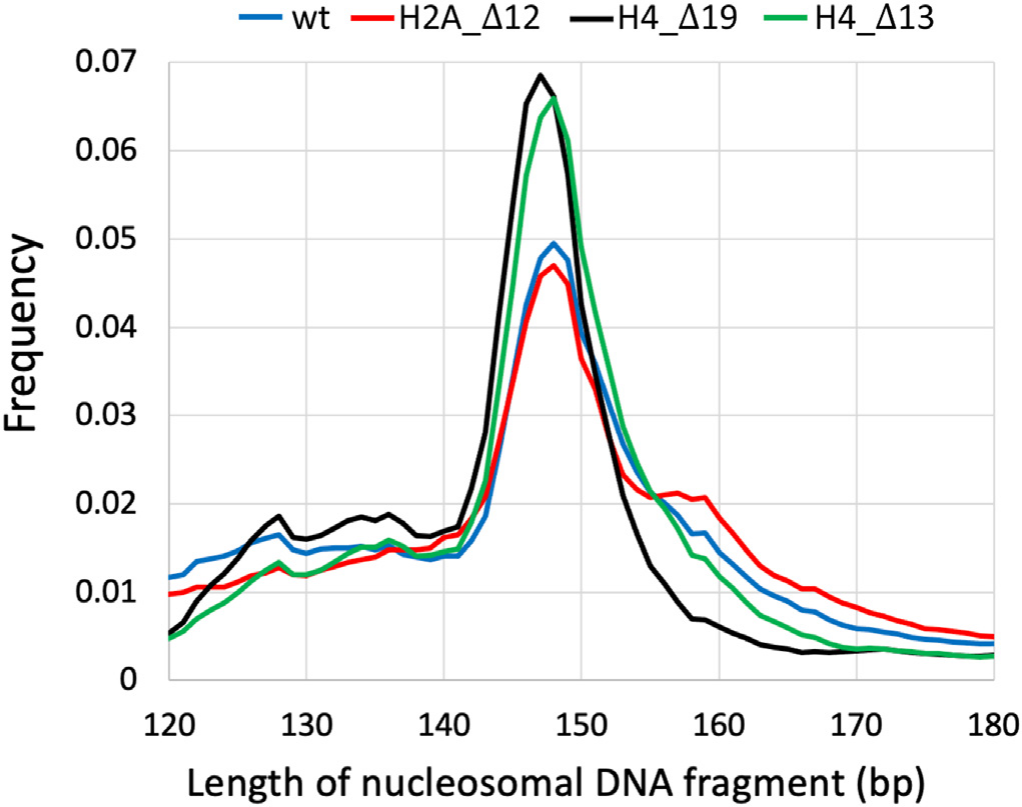
**Distributions of the lengths of nucleosomes reconstituted with the wild type (wt) and H2A/H4 N-tailless histones.** To analyze distributions of mono- and di-nucleotides in nucleosomes, the DNA fragments with the following lengths were selected: 147 to 149 bp for wt, H2A_Δ12 and H4_Δ13 datasets, and 146 to 148 bp for H4_Δ19 dataset. The numbers of selected fragments were: 18 mil for wt, 17 mil for H2A_Δ12, 31 mil for H4_Δ13 and 27 mil for H4_Δ19 datasets.

Reconstituted chromatin (1 μg in 60 μl) in buffer containing 50 mM NaCl, 10 mM Tris 0.025% NP-40, 20% Ficoll, 0.7 mM CaCl_2_ was digested at different micrococcal nuclease (MNase) concentrations. Digested material was run on 16% acrylamide gel with molecular length marker. Digestion conditions (0.2 units MNase for 10 min at 20 °C) that produced 147 to 148 bp-long predominant band were chosen to perform large scale digestion (Fig. 7). Reaction was stopped by addition of 20 mM EDTA and cooled on ice. Digested samples were treated with SDS and proteinase K for 2 h at 55 °C and cleaned with Promega Wizard SV Gel and PCR Clean-Up System (cat. # A9282). Nucleosomal DNA fragments were subjected to paired-end sequencing on Illumina platform.

### *In vitro* nucleosome fragments

Raw FASTQ read pairs were trimmed with *cutadapt* (58). Trimmed read pairs were assembled with *PEAR* (59) to obtain nucleosome-size fragments. *p*-value for *PEAR* was set as *-p* 0.0001. The read pairs that failed to assemble with this *p*-value were discarded. The resulting fragments were then mapped with Bowtie2 (37, parameter -*x*), using the sequence of the *BRCA1* gene (from human genome hg19) as a reference. Fragments with *samtools* (60) flag 260 (unmapped/not primary alignment) were filtered out. To calculate mono- and di-nucleotide frequency profiles in nucleosomes, we selected DNA fragments with the lengths corresponding to the peaks of distributions presented in Figure 7: 147 to 149 bp for wt, H2A_Δ12 and H4_Δ13 datasets, and 146 to 148 bp for H4_Δ19 dataset.

### *In vivo* nucleosome datasets

Several nucleosomal DNA datasets obtained *in vivo* were used in this study. Two datasets, one from yeast (61) and the other from mouse (62), were generated by the chemical method, and the dyad positions were precisely mapped at the single base-pair resolution. The other datasets were produced by paired-end MNase-seq from yeast (63), *Drosophila* S2 cells (64), mouse embryonic stem cells (62), and human lymphoblastoid cell lines GM18508 and GM19238 (65). BAM files were either downloaded from NCBI GEO database or obtained by mapping raw reads to the corresponding genomes using the default settings of Bowtie two (66), *i.e*., --sensitive, -I 0, -X 500. Only the fragments of 147 bp in length were used to calculate mono- and di-nucleotide frequency profiles *in vivo*.

## Data availability

Processed sequencing data generated in this study have been submitted to the NCBI Gene Expression Omnibus (GEO; http://www.ncbi.nlm.nih.gov/geo/) under accession number GSE252427.

## Supporting information

This article contains supporting information (Figs. S1–S5).

## Conflict of interest

The authors declare that they have no conflicts of interest with the contents of this article.
