# Supplementary material for: Histone N-tails modulate sequence-specific positioning of nucleosomes[image]
Source: J Biol Chem. 2024 Dec 26;301(2):108138. doi: 10.1016/j.jbc.2024.108138 (PMC11803869; doi:10.1016/j.jbc.2024.108138)
Supplement: Supporting information [file mmc1.pdf]

Supporting Information

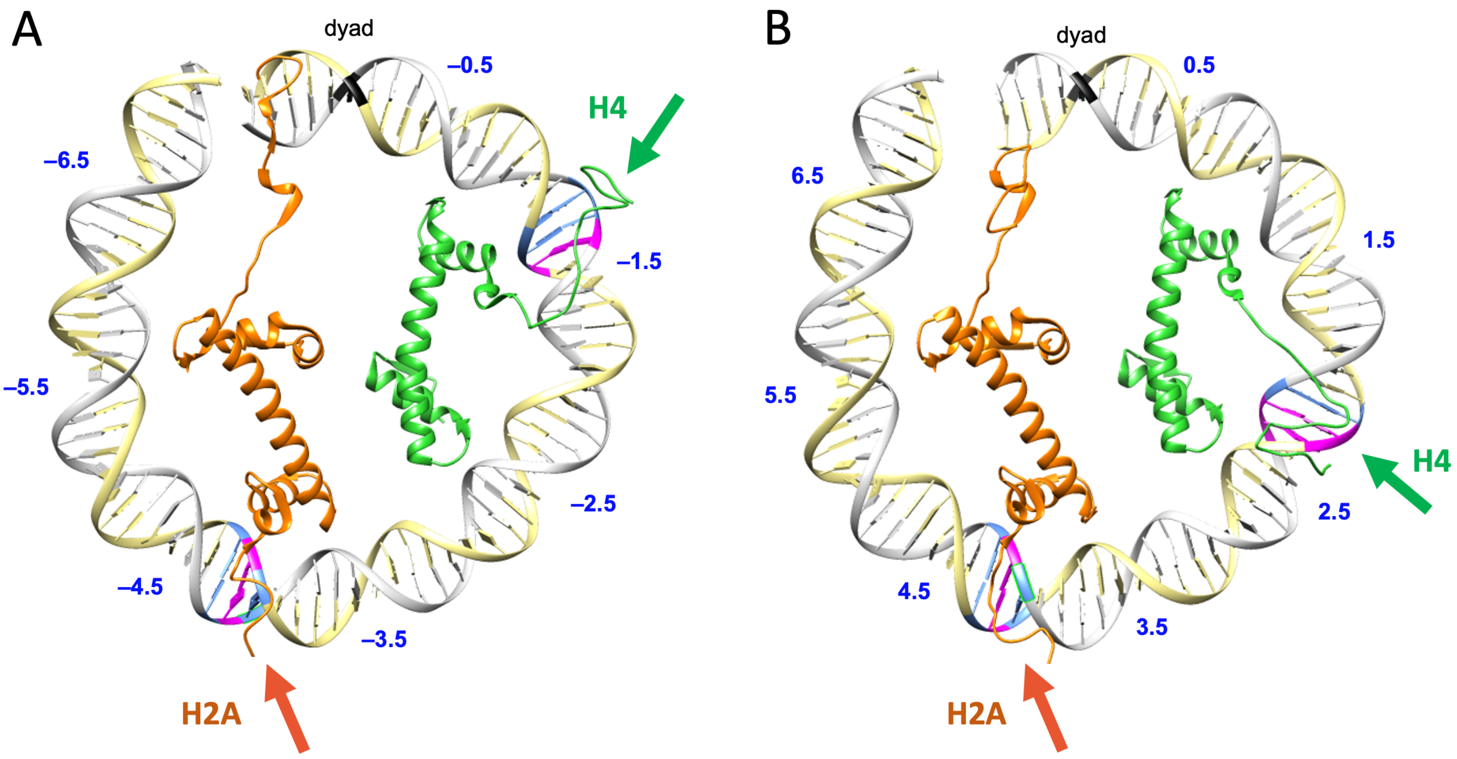

Supporting Figure S1. Histone H2A and H4 N-tails in the DNA minor grooves.

The ventral (A) and dorsal (B) halves of the nucleosome X-ray crystal structure (32) are shown schematically: the DNA is given in ribbon representation with the dyad colored in black. Only histones H2A (orange) and H4 (green) are shown, whereas the other histones are hidden for clarity. The superhelical locations of DNA (SHL) are numbered from -6.5 to -0.5 in (A) and from 0.5 to 6.5 in (B). The nucleotides in close vicinity of H2A and H4 N-tails are colored in blue (A:T pairs) and in magenta (G:C pairs). Note the difference between the H2A and H4 tails – in both halves of nucleosome, the H2A N-tails interact with the DNA minor grooves close to SHL -4/4, while the H4 N-tails penetrate minor grooves between SHL -1 and -1.5 in (A) and between SHL 2 and 2.5 in (B); see the orange and green arrows. This delocalization of the H4 N-tail may explain its broader effect on the nucleosome positioning, compared to the local effect of the H2A N-tail (see Figures 3B and 4B).

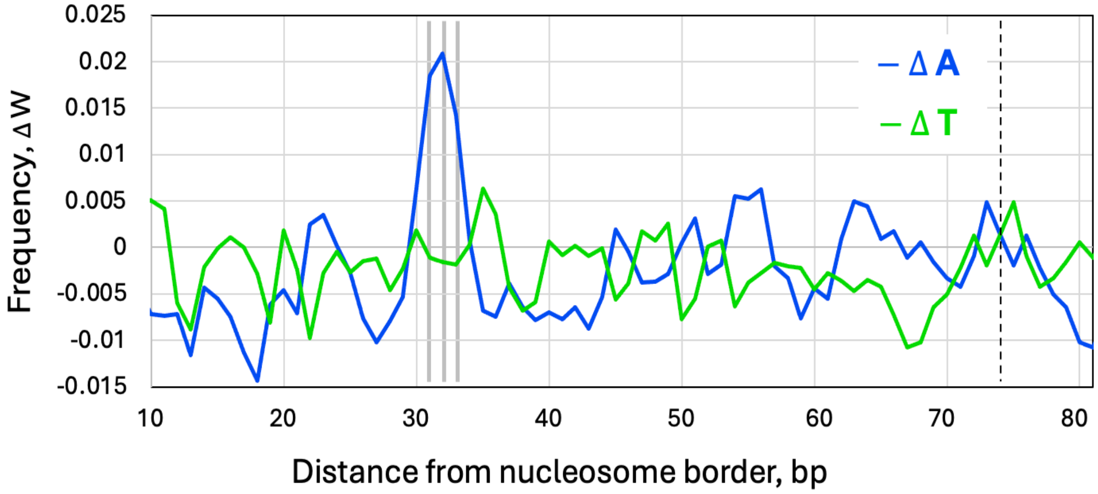

Supporting Figure S2. Statistically significant difference in the fraction of adenines between the wt- and H2A_Δ12-sets of nucleosomes. See Figure 3 for notations.

We generated 1,000 random, non-overlapping subsets, each consisting of 10,000 sequencing reads, to analyze nucleotide proportions at each position. For each subset, we computed the fractions of A and T across all positions. These fractions were symmetrized, where the symmetrized fraction for adenine (Sym_A) was calculated as the average of the fraction of A and the reverse complement fraction of T, i.e., Sym_A = (fraction_A + rev_fraction_T) / 2. This process yielded a distribution of 1,000 values for each nucleotide at each position. To assess significant differences in nucleotide fraction distributions across nucleosomal positions, the paired t-tests were performed. The p-values from these tests were adjusted using Bonferroni correction, with a significance threshold set at p-adjusted < 0.001. The grey vertical lines indicate positions #31-33 satisfying this criterium in the case of adenine (with the difference between the means being greater than 1%).

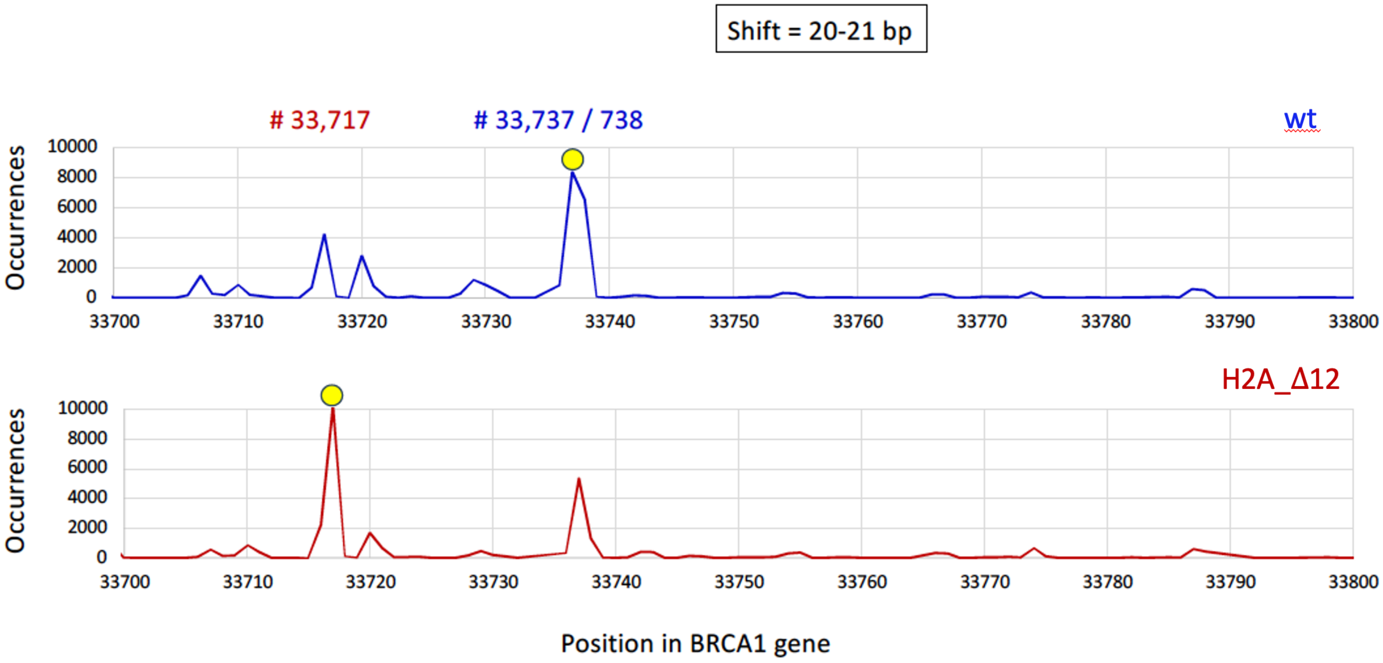

Supporting Figure S3. A representative region of BRCA1 gene where the nucleosome occurrences have two strong peaks separated by 20-21 bp.
The predominant NCP positions in the wt and H2A_Δ12 populations of nucleosomes are shifted by 20-21 bp one from the other, so that the two NCP positions are in the same phase.

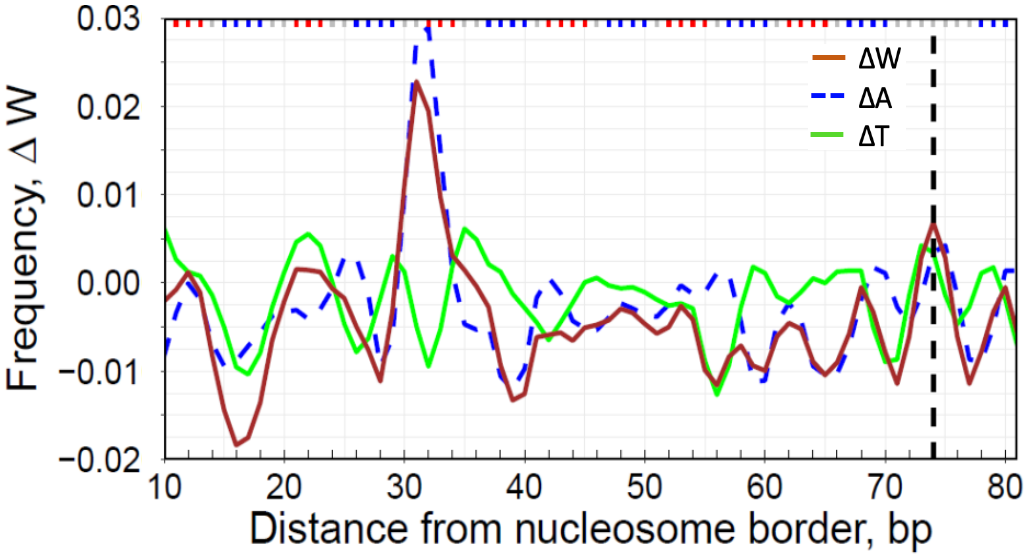

Supporting Figure S4. Statistically significant difference in the fraction of adenines between the wt- and H2A_Δ12-sets of nucleosomes.

DNA sequence analysis of nucleosomes reconstituted with the H2A N-tailless histones and digested simultaneously with MNase and exonuclease III (46, 48). The differential ΔW, ΔA and ΔT profiles are shown in brown, broken blue and green lines, respectively (compare with Figure 3B). Importantly, the combined MNase and ExoIII cleavage largely eliminates the MNase-associated WW and SS peaks at the boundary of the nucleosome core (48). Note that the MNase and MNase-Exo data are entirely consistent: Similarly to the MNase profiles presented in Figure 3B, the ΔW and ΔA profiles of the MNase-Exo data are characterized by strong peaks at positions #31-32 (where the H2A tail interacts with the DNA minor groove).

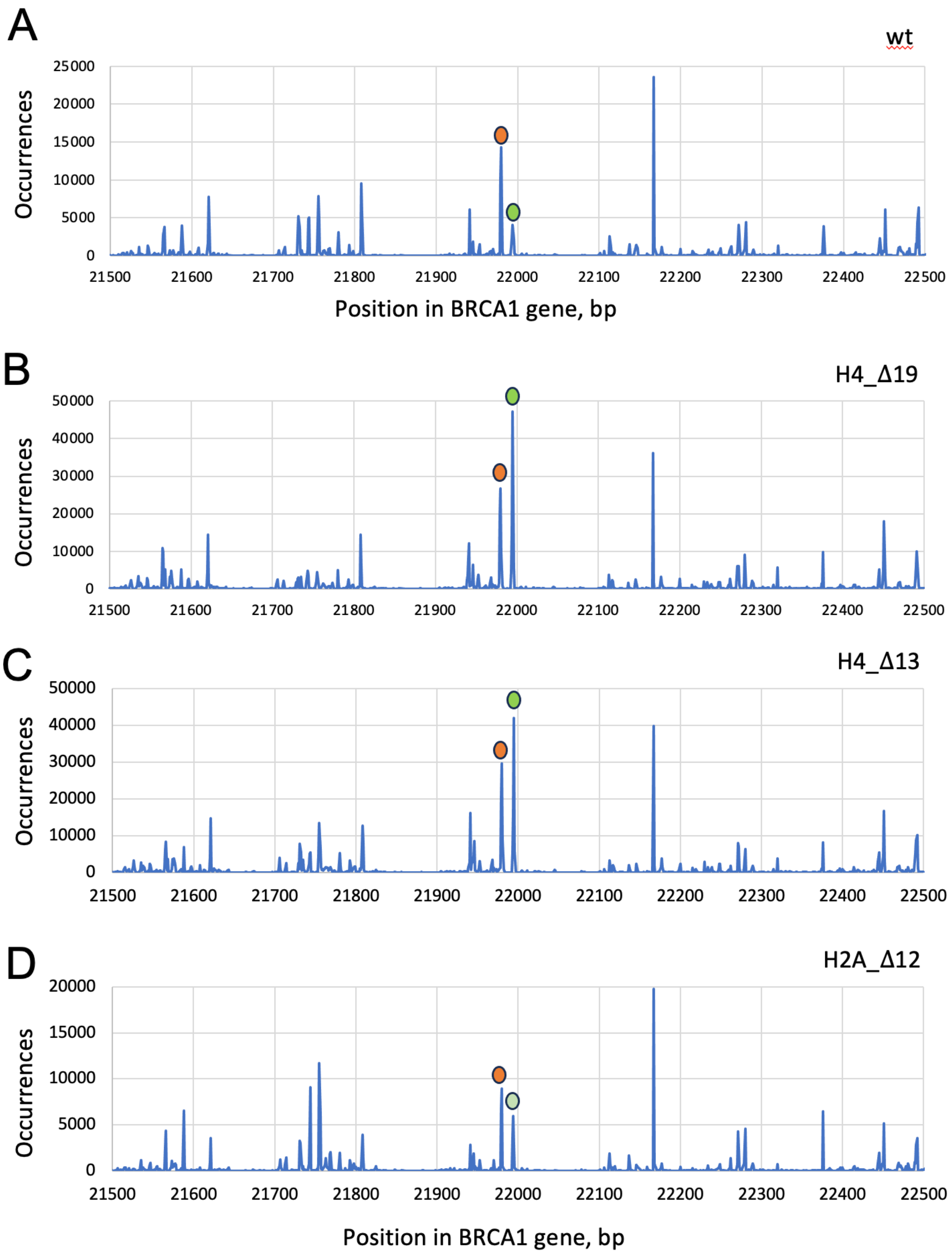

Supporting Figure S5. A representative region of BRCA1 gene where the nucleosome occurrences have two strong peaks separated by 15 bp.

The nucleosome occurrences in the region 21,500 - 22,500 bp of BRCA1 gene are presented for the four NCP subsets (wt, H4_Δ19, H4_Δ13 and H2A_Δ12). Note that in the wt and H2A_Δ12 subsets, the nucleosomes shown in red represent the dominant NCP positions, whereas in the H4_Δ19 and H4_Δ13 subsets, these are the positions shown in green that are dominant. Intriguingly, in the region 21,500 - 22,200 bp there are four peak clusters separated by 190-200 bp, which is remarkably close to the nucleosome spacing *in vivo*. This periodicity does not hold for the whole BRCA1 gene, however (data not shown).
